# Development and validation of a novel nomogram for predicting outcomes in advanced lung cancer patients treated beyond progression with immune checkpoint inhibitors

**DOI:** 10.3389/fimmu.2025.1643591

**Published:** 2025-11-10

**Authors:** Tianwei Luo, Haiyun Geng, Genwang Wang, Ying Zhao, Ning Han, Ting Ouyang, Ao Chen, Xiufeng Liu, Chao Chen, Mi Yang

**Affiliations:** 1 Department of Oncology, Nanjing Drum Tower Hospital, Nanjing Drum Tower Hospital Clinical College, Nanjing University of Chinese Medicine, Nanjing, China; 2 Department of Oncology, Jinling Hospital, Medical School of Nanjing University, Nanjing, China; 3 Department of Medical Administration, Jinling Hospital, Medical School of Nanjing University, Nanjing, China; 4 Department of Radiology, Jinling Hospital, Nanjing Medical University, Nanjing, China; 5 Department of Oncology, Jinling Hospital, Nanjing Medical University, Nanjing, China

**Keywords:** lung cancer (LC), immunotherapy, immune checkpoint inhibitors (ICIs), treatment beyond progression (TBP), nomogram prognostic model

## Abstract

**Background:**

Standardized management of lung cancer (LC) progressing post-immunotherapy remains challenging, with debated clinical benefits of treatment beyond progression (TBP). We evaluated the efficacy of continued immune checkpoint inhibitors (ICIs) and developed a pharmacologically guided nomogram to optimize TBP decision-making.

**Methods:**

This retrospective analysis of 153 LC patients undergoing post-progression ICIs continuation identified significant predictors via the least absolute shrinkage and selection operator (LASSO) regression and multivariate Cox analysis. A nomogram predicting overall survival (OS) and progression-free survival (PFS) was constructed and validated using time-dependent receiver operating characteristic (ROC) curves, calibration plots, and decision curve analysis (DCA).

**Results:**

Treatment regimen, Eastern Cooperative Oncology Group (ECOG) performance status, efficacy evaluation, lymph node metastasis, and liver metastasis independently predicted OS, while liver metastasis and efficacy evaluation influenced PFS. The nomogram demonstrated robust discrimination (OS C-index=0.700; PFS C-index=0.599) with area under the curve (AUC) values of 0.786/0.777/0.705 (OS) and 0.621/0.638/0.630 (PFS) at 6/12/24 months. DCA confirmed the clinical utility of the OS nomogram. Patients were stratified into high- and low-risk groups based on optimal cutoff values, and risk stratification revealed significant survival differences (p<0.01).

**Conclusion:**

This validated nomogram provides a clinically actionable tool to identify patients benefiting from sustained ICIs exposure, enabling pharmacologically informed TBP strategies while minimizing futile drug exposure. Future prospective multicenter studies should validate utility across diverse pharmacogenomic populations.

## Introduction

Lung cancer (LC) is among the most prevalent and deadly malignancies worldwide, with non-small-cell lung cancer (NSCLC) accounting for approximately 85% of cases. While patients with stage IB NSCLC have a five-year overall survival (OS) rate of up to 68%, those with advanced-stage (IVA-IVB) disease have dismal rates of just 0–10% (1). Rising tobacco consumption, especially in Asia, threatens to further increase mortality (2).

The advent of immune checkpoint inhibitors (ICIs) has significantly improved survival in some NSCLC patients. By restoring T-cell function, ICIs enhance the immune system’s antitumor activity and have become a cornerstone in the treatment of advanced NSCLC (3, 4). However, most patients eventually experience disease progression (PD), and the optimal treatment strategy post-progression remains contentious. Traditional imaging criteria (e.g., RECIST) often inadequately assess delayed pharmacodynamic responses to ICIs, including pseudoprogression or hyperprogression, potentially prompting inappropriate therapy discontinuation in responsive patients (5). Unlike traditional chemoradiation, immunotherapy’s atypical response patterns complicate clinical assessment (6).

The clinical benefits of treatment beyond progression (TBP) with ICIs are still debated. Some clinical trials and real-world studies have reported that TBP can significantly improve OS and progression-free survival (PFS) (7–9). For example, Reinhorn et al. (10) demonstrated that 36% of patients receiving TBP achieved clinical benefit, with 27% maintaining a progression-free interval exceeding six months. However, other studies have failed to confirm significant survival advantages with TBP (11, 12). Current NCCN guidelines (v4.2024) stratify post-progression management by driver mutations, progression pattern (oligo- vs. polyprogression), and programmed cell death ligand 1 (PD-L1) status (13). For oligoprogressive disease (≤5 lesions, no new symptoms), local therapies combined with ICIs maintenance are recommended. For extensive progression, strategies include platinum-chemotherapy/anti-angiogenics or ICIs rechallenge combinations. Clinical trials exploring novel agents (e.g., TROP2-ADCs, TGF-β/PD-L1 bispecifics) are prioritized, while targeted therapies remain first-line for oncogene-driven NSCLC.

However, the clinical utility of these strategies remains limited by tumor heterogeneity. Pharmacologically, the decision to continue ICIs therapy beyond progression hinges on balancing sustained antitumor immune activation against cumulative toxicity risks from prolonged drug exposure. Current clinical guidelines lack tools to quantify this trade-off, particularly for patients with low PD-L1 expression or driver-negative tumors who derive limited benefit from conventional biomarkers. A dynamic predictive model capable of stratifying patients by their likelihood of benefit from extended ICIs exposure is thus urgently needed to optimize pharmacological decision-making. Our nomogram addresses this gap by integrating immune-microenvironmental determinants (e.g., liver metastasis-induced tolerance) and treatment response patterns to guide precision in ICIs dosing duration, minimizing futile drug exposure while maximizing survival gains.

## Methods

### Study design and patient selection

This retrospective study analyzed 461 lung cancer (LC) patients who received immune checkpoint inhibitors (ICIs) therapy at Nanjing Jinling Hospital between January 2019 and February 2024. Patients were included in the study based on the following criteria:

- Pathologically confirmed diagnosis of LC;
- Treatment with ICIs as monotherapy or in combination with other therapies;
- At least two cycles of ICIs therapy completed prior to disease progression (PD);
- Continuation of ICIs therapy after PD;
- Presence of at least one measurable lesion as defined by the Response Evaluation Criteria in Solid Tumors (RECIST) version 1.1;
- Investigator-assessed potential clinical benefit from continued ICIs therapy.

#### Exclusion criteria

Patients meeting any of the following criteria were excluded:

- Diagnosis of other concurrent malignancies;
- Discontinuation of treatment due to intolerable toxicity or non-drug-related reasons;
- Incomplete clinical or follow-up data.

### Follow-up and ethical approval

After applying the inclusion and exclusion criteria, a total of 153 patients were included in the final analysis. The last follow-up date was April 29, 2024. The study was conducted in accordance with the Declaration of Helsinki (revised in 2013) and was approved by the institutional ethics committee. Written informed consent was obtained from all participants.

### Clinical evaluation and baseline characteristics

Radiological assessments were jointly performed by oncologists and radiologists. The initial evaluation of treatment response was typically conducted after two treatment cycles or earlier if deemed necessary based on clinical judgment. Efficacy assessment was defined as the best objective response achieved during the initial ICIs treatment period, evaluated according to RECIST v1.1 criteria.

The baseline clinical characteristics collected included patient demographics, treatment history, and laboratory parameters, as follows:

- Demographic and medical history: Age, gender, smoking status, prior treatments, histological subtypes of lung cancer, and clinical staging.
- Tumor characteristics: Gene mutations, Eastern Cooperative Oncology Group (ECOG) performance status, and metastatic sites (e.g., liver, lymph nodes, bones).
- Treatment details: Types of ICIs (e.g., Camrelizumab, Sintilimab), treatment regimens (monotherapy or combination therapy), and clinical response evaluation (stable disease [SD], disease progression [PD], or partial remission [PR]).
- Laboratory indicators: White blood cell (WBC) count, hemoglobin (HGB), neutrophil to lymphocyte ratio (NLR), platelet to lymphocyte ratio (PLR), and tumor markers (CEA, CA125, and CA199).

### Study endpoints

The primary endpoints of the study were overall survival (OS) and progression-free survival (PFS), defined as follows:

- Overall survival (OS): The time from the first progression following ICIs therapy to death from any cause.
- Progression-free survival (PFS): The time from the first progression following ICIs therapy to subsequent progression, relapse, or death from any cause, as determined at the most recent follow-up.

### Safety assessments

All eligible patients underwent safety evaluations, and the severity of adverse events was graded according to version 5.0 of the Common Terminology Criteria for Adverse Events (CTCAE) established by the National Cancer Institute.

### Statistical analysis

#### Variable selection and model construction

To identify significant prognostic factors for OS and PFS, the least absolute shrinkage and selection operator (LASSO) regression method was applied to select key variables. Multivariate Cox proportional hazards regression analysis was then used to evaluate the independent associations between these variables and patient outcomes. Based on the results, a nomogram was constructed to predict OS and PFS at 6, 12, and 24 months. Based on risk scores derived from the OS and PFS nomograms, the optimal cutoff value was determined using the “surv_cutpoint” function in the “survminer” R package, stratifying patients into high- and low-risk groups, with subsequent intergroup survival differences analyzed by the Kaplan-Meier method.

#### Model evaluation

The predictive performance of the nomogram was assessed using three approaches:

- Discrimination: Time-dependent receiver operating characteristic (ROC) curves were used to calculate the area under the curve (AUC), which reflects the model’s ability to distinguish between different survival outcomes.
- Calibration: Calibration plots were constructed to compare the predicted survival probabilities with actual survival rates, thereby evaluating the agreement between predicted and observed values. To address calibration drift from limited sample size, internal validation was performed via 1,000 bootstrap resampling iterations, correcting for potential overfitting bias.
- Clinical Utility: Decision curve analysis (DCA) was conducted to assess the net benefit of the model at different threshold probabilities, demonstrating its clinical applicability.

#### Statistical software and thresholds

All statistical analyses were performed using R software (version 4.1.3). Continuous variables were expressed as means ± standard deviation or medians (interquartile range), and categorical variables were presented as frequencies and percentages. Group differences were analyzed using the paired t-test for continuous variables or Mann–Whitney U test for nonparametric data and the χ² test or Fisher’s exact test for categorical variables. A two-sided p-value < 0.05 was considered statistically significant.

## Result

### Patient characteristics

**Figure 1:**
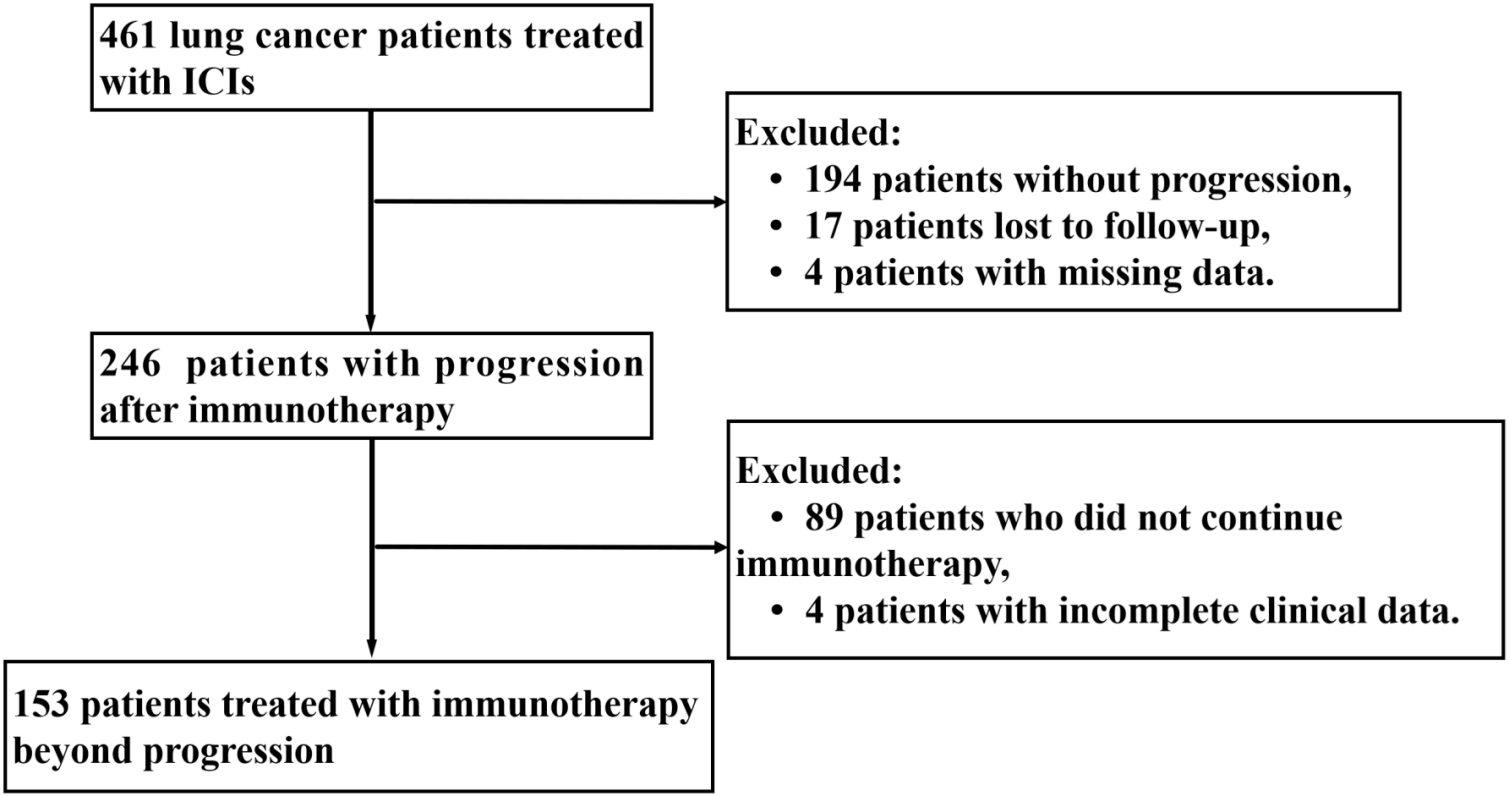
Study flowchart. LC, lung cancer; ICIs, immune checkpoint inhibitors.

From the initiation of immunotherapy to the last follow-up date, **Figure 1** shows that 153 patients with TBP were evaluated according to specified criteria. The cohort consisted of 126 males and 27 females; 55.56% (85) had a smoking history, and 76.47% (117) were under 70 years old. Histologic subtypes were distributed as follows: 48.37% (74) lung adenocarcinoma, 37.25% (57) small cell carcinoma, and 12.42% (19) squamous carcinoma. According to the eighth edition of the TNM classification, 22.88% (35) were diagnosed with stage II-III and 77.12% (118) with stage IV. Immunotherapy was administered as first-line treatment for 93 patients (60.78%), with 139 patients (90.85%) having a baseline ECOG performance status of 0 or 1. Initially, a minority of patients presented with multiple metastases: lung (38.56%), bone (20.92%), liver (13.73%), and lymph node (24.84%). The primary immunologic drugs used were camrelizumab (33.33%), pembrolizumab (5.88%), sintilimab (17.65%), and trelizumab (30.07%). Immunologic combination regimens comprised ICIs with chemotherapy (70.59%), anti-vascular therapies (10.46%), both chemotherapy and anti-vascular therapies (11.11%), and ICI monotherapy (7.84%). Post-immunotherapy, 60.78% (93) of patients achieved disease control (stable disease [SD] or partial remission [PR]), while 52.29% (80) presented with grade 1–2 immune-related adverse events (irAEs), primarily immune inflammation such as pneumonia, dermatitis, hepatitis. Further details on baseline clinical variables are provided in **Table 1**.

**Table 1:** Clinical baseline characteristics of lung cancer patients.

Variables	Total (n = 153)	Dead (n = 78)	Alive (n = 75)	P	χ²/Fisher
Age (year)				0.413	0.670
≤70	117 (76.47)	57 (73.08)	60 (80)		
>70	36 (23.53)	21 (26.92)	15 (20)		
CEA (ng/mL)				0.492	0.472
≤9.8	54 (35.29)	25 (32.05)	29 (38.67)		
>9.8	99 (64.71)	53 (67.95)	46 (61.33)		
CA125 (U/mL)				0.941	0.005
≤35	32 (20.92)	17 (21.79)	15 (20)		
>35	121 (79.08)	61 (78.21)	60 (80)		
CA199 (U/mL)				0.280	1.167
≤37	69 (45.1)	39 (50)	30 (40)		
>37	84 (54.9)	39 (50)	45 (60)		
WBC (×10^9^/L)				0.243	1.363
≤3.5	15 (9.8)	5 (6.41)	10 (13.33)		
>3.5	138 (90.2)	73 (93.59)	65 (86.67)		
HGB (g/L)				0.999	<0.001
≤115	54 (35.29)	28 (35.9)	26 (34.67)		
>115	99 (64.71)	50 (64.1)	49 (65.33)		
NLR				0.711	0.137
≤5.06	107 (69.93)	53 (67.95)	54 (72)		
>5.06	46 (30.07)	25 (32.05)	21 (28)		
PLR				0.305	1.051
≤205.99	89 (58.17)	49 (62.82)	40 (53.33)		
>205.99	64 (41.83)	29 (37.18)	35 (46.67)		
Types of ICIs drugs				0.017	Fisher
Camrelizumab	51 (33.33)	22 (28.21)	29 (38.67)		
Pembrolizumab	9 (5.88)	4 (5.13)	5 (6.67)		
Sintilimab	27 (17.65)	12 (15.38)	15 (20)		
Trelizumab	46 (30.07)	33 (42.31)	13 (17.33)		
Others	20 (13.07)	7 (8.97)	13 (17.33)		
Gender				0.337	0.923
Male	126 (82.35)	67 (85.9)	59 (78.67)		
Female	27 (17.65)	11 (14.1)	16 (21.33)		
Smoking history				0.786	0.074
No	68 (44.44)	36 (46.15)	32 (42.67)		
Yes	85 (55.56)	42 (53.85)	43 (57.33)		
Surgery				0.293	1.108
No	120 (78.43)	58 (74.36)	62 (82.67)		
Yes	33 (21.57)	20 (25.64)	13 (17.33)		
Previous radiotherapy				0.385	0.756
No	121 (79.08)	59 (75.64)	62 (82.67)		
Yes	32 (20.92)	19 (24.36)	13 (17.33)		
Chemotherapy				0.575	0.315
No	78 (50.98)	42 (53.85)	36 (48)		
Yes	75 (49.02)	36 (46.15)	39 (52)		
Targeted				0.999	<0.001
No	134 (87.58)	68 (87.18)	66 (88)		
Yes	19 (12.42)	10 (12.82)	9 (12)		
Anti-vascular				0.458	0.551
No	125 (81.7)	66 (84.62)	59 (78.67)		
Yes	28 (18.3)	12 (15.38)	16 (21.33)		
Pathology				0.051	Fisher
Adenocarcinoma	74 (48.37)	34 (43.59)	40 (53.33)		
Small cell carcinoma	57 (37.25)	36 (46.15)	21 (28)		
Squamous cell carcinoma	19 (12.42)	6 (7.69)	13 (17.33)		
Others	3 (1.96)	2 (2.56)	1 (1.33)		
Staging				0.524	0.407
II-III	35 (22.88)	20 (25.64)	15 (20)		
IV	118 (77.12)	58 (74.36)	60 (80)		
Gene mutation				0.690	0.742
No	37 (24.18)	17 (21.79)	20 (26.67)		
Yes	19 (12.42)	9 (11.54)	10 (13.33)		
Unknow	97 (63.4)	52 (66.67)	45 (60)		
Immunization combination regimen				0.408	2.894
ICIs + chemotherapy	108 (70.59)	56 (71.79)	52 (69.33)		
ICIs + chemotherapy + anti-vascular	17 (11.11)	6 (7.69)	11 (14.67)		
ICIs + anti-vascular	16 (10.46)	8 (10.26)	8 (10.67)		
ICIs monotherapy	12 (7.84)	8 (10.26)	4 (5.33)		
Line				0.969	0.063
1	93 (60.78)	47 (60.26)	46 (61.33)		
2	36 (23.53)	19 (24.36)	17 (22.67)		
>2	24 (15.69)	12 (15.38)	12 (16)		
ECOG PS				0.358	0.843
0~1	139 (90.85)	73 (93.59)	66 (88)		
>2	14 (9.15)	5 (6.41)	9 (12)		
Efficacy assessment				< 0.001	13.958
PD	60 (39.22)	20 (25.64)	40 (53.33)		
SD	67 (43.79)	39 (50)	28 (37.33)		
PR	26 (16.99)	19 (24.36)	7 (9.33)		
irAEs score				0.444	1.624
0	62 (40.52)	30 (38.46)	32 (42.67)		
1~2	80 (52.29)	44 (56.41)	36 (48)		
3+	11 (7.19)	4 (5.13)	7 (9.33)		
Ascites				0.856	0.033
No	137 (89.54)	69 (88.46)	68 (90.67)		
Yes	16 (10.46)	9 (11.54)	7 (9.33)		
Lung metastasis				0.072	3.245
No	94 (61.44)	42 (53.85)	52 (69.33)		
Yes	59 (38.56)	36 (46.15)	23 (30.67)		
Liver metastasis				0.300	1.075
No	132 (86.27)	70 (89.74)	62 (82.67)		
Yes	21 (13.73)	8 (10.26)	13 (17.33)		
Bone metastasis				0.263	1.252
No	121 (79.08)	65 (83.33)	56 (74.67)		
Yes	32 (20.92)	13 (16.67)	19 (25.33)		
Lymph node metastasis				0.744	0.107
No	115 (75.16)	60 (76.92)	55 (73.33)		
Yes	38 (24.84)	18 (23.08)	20 (26.67)		
Other metastasis				0.483	0.491
No	115 (75.16)	61 (78.21)	54 (72)		
Yes	38 (24.84)	17 (21.79)	21 (28)		
Radiotherapy				0.600	0.275
No	94 (61.44)	50 (64.1)	44 (58.67)		
Yes	59 (38.56)	28 (35.9)	31 (41.33)		

Data is expressed as n(%). CEA, carcinoembryonic antigen; CA125, carbohydrate antigen 125; CA199, carbohydrate antigen 199; WBC, white blood cell; HGB, hemoglobin; NLR, neutrophil to lymphocyte ratio; PLR, platelet to lymphocyte ratio; ICIs, immune checkpoint inhibitors; TBP, treatment beyond progression; Gene mutation: No, means non-exist EGFR/ALK/ROS1 gene mutation; Yes, means exist EGFR/ALK/ROS1 mutation; Unknown is no check; ECOG PS, Eastern Cooperative Oncology Group performance status; PD, disease progression; SD, stable disease; PR, partial remission; irAEs, immune-related adverse events.

### LASSO and multivariate Cox regression analysis

**Figure 2:**
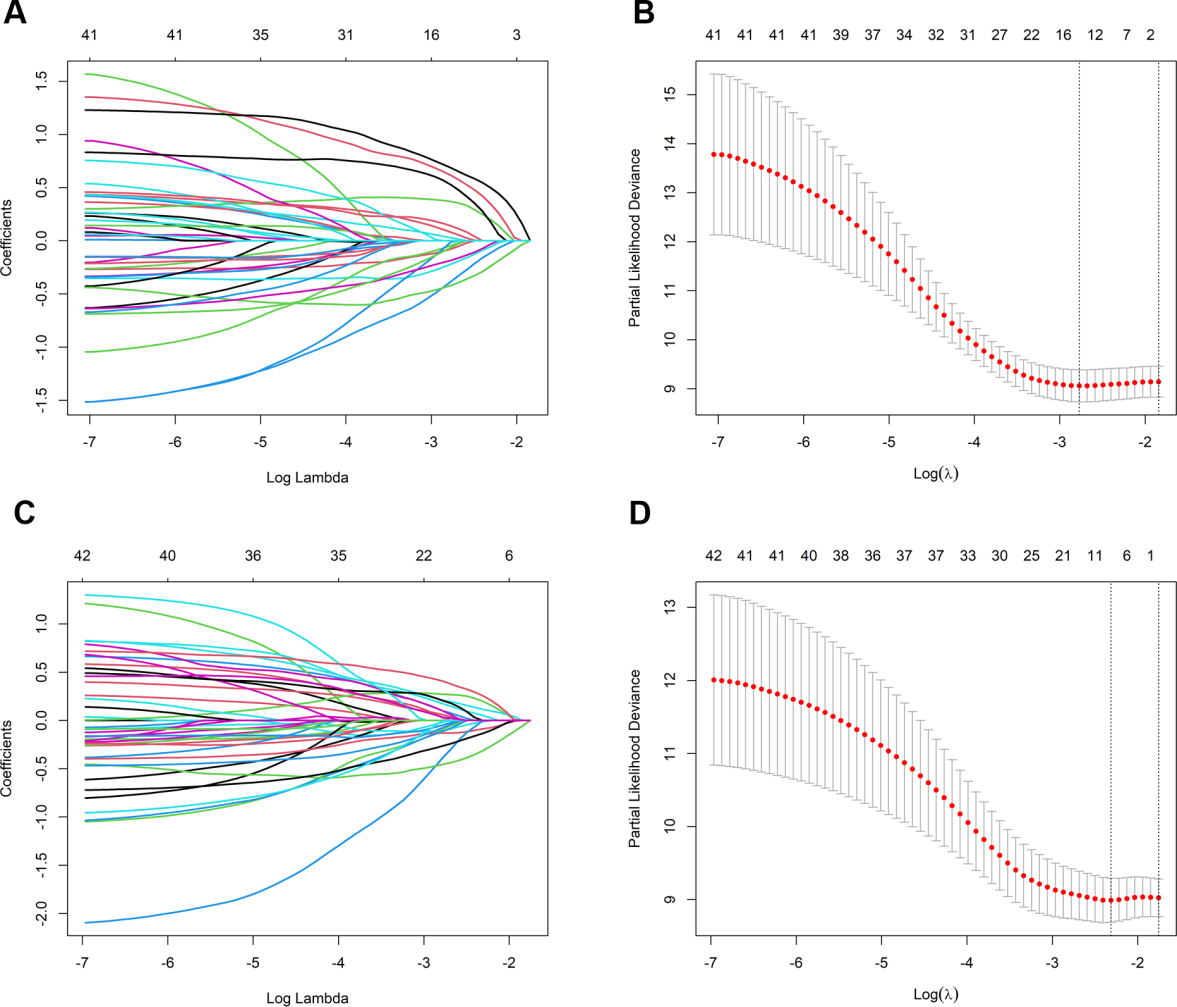
Predictor selection for OS and PFS using LASSO regression. **(A)** LASSO coefficient trajectories for OS predictors; **(B)** Ten-fold cross-validation for OS; selection of optimal penalty parameter (λ); **(C)** LASSO coefficient trajectories for PFS predictors; **(D)** Ten-fold cross-validation for PFS: selection of optimal penalty parameter (λ). OS, overall survival; PFS, progression-free survival; LASSO, least absolute shrinkage and selection operator.

Prognostic models were developed by screening 31 clinical factors through LASSO regression with 10-fold cross-validation (**Figures 2A, B**). Independent risk factors for OS were then assessed using multivariate Cox regression analysis (**Table 2**). The results indicated that immunological combination regimens, ECOG performance status, efficacy assessment, lymph node, and liver metastasis strongly predicted OS in LC patients (p<0.050), with efficacy assessment and lymph node metastasis as protective factors (HR<1) and the others as risk factors. These variables were integrated into an OS nomogram for individualized risk quantification. For PFS prediction, LASSO-Cox analysis identified liver metastasis and efficacy assessment as key prognosticators (**Figures 2C, D**, **Table 3**).

**Table 2:** Multivariate Cox regression analyses for OS.

Variables	HR	95%CI	P	Wald	B	SE
CEA (ng/mL)						
≤9.8	Ref					
>9.8	0.760	0.458-1.260	0.287	1.134	-0.275	0.258
Types of ICIs drugs						
Camrelizumab	Ref					
Pembrolizumab	2.666	0.742-9.586	0.133	2.256	0.981	0.653
Sintilimab	1.353	0.660-2.773	0.409	0.681	0.302	0.366
Trelizumab	0.707	0.331-1.510	0.370	0.803	-0.347	0.387
Others	1.141	0.465-2.801	0.773	0.083	0.132	0.458
Smoking history						
No	Ref					
Yes	1.575	0.929-2.669	0.092	2.844	0.454	0.269
Surgery						
No	Ref					
Yes	0.533	0.242-1.174	0.118	2.442	-0.630	0.403
Pathology						
Adenocarcinoma	Ref					
Small cell carcinoma	0.792	0.396-1.581	0.508	0.438	-0.234	0.353
Squamous cell carcinoma	1.480	0.618-3.548	0.379	0.774	0.392	0.446
Others	0.363	0.043-3.037	0.350	0.873	-1.012	1.083
Staging						
II-III	Ref					
IV	1.103	0.558-2.182	0.778	0.079	0.098	0.348
Immunization combination regimen						
ICIs + chemotherapy	Ref					
ICIs + chemotherapy + anti-vascular	2.621	1.165-5.896	0.020	5.427	0.964	0.414
ICIs + anti-vascular	1.145	0.507-2.586	0.744	0.107	0.136	0.416
ICIs monotherapy	0.329	0.097-1.117	0.075	3.180	-1.113	0.624
ECOG PS						
0~1	Ref					
>2	2.692	1.213-5.976	0.015	5.922	0.990	0.407
Efficacy assessment						
PD	Ref					
SD	0.727	0.414-1.278	0.268	1.226	-0.319	0.288
PR	0.412	0.172-0.985	0.046	3.978	-0.887	0.445
Lung metastasis						
No	Ref					
Yes	0.597	0.327-1.090	0.093	2.820	-0.516	0.307
Liver metastasis						
No	Ref					
Yes	2.925	1.360-6.290	0.006	7.552	1.073	0.391
Bone metastasis						
No	Ref					
Yes	1.560	0.845-2.879	0.155	2.023	0.445	0.313
Lymph node metastasis						
No	Ref					
Yes	0.538	0.291-0.995	0.048	3.902	-0.619	0.313

HR, hazard ratio; CI, confidence interval; SE, standard error; Ref, reference; CEA, carcinoembryonic antigen; ICIs, immune checkpoint inhibitors; ECOG PS, Eastern Cooperative Oncology Group performance status; PD, disease progression; SD, stable disease; PR, partial remission.

**Table 3:** Multivariate Cox regression analyses for PFS.

Variables	HR	95%CI	P	Wald	B	SE
Types of ICIs drugs						
Camrelizumab	Ref					
Pembrolizumab	0.999	0.393-2.540	0.998	0.000	-0.001	0.476
Sintilimab	0.746	0.398-1.398	0.360	0.838	-0.294	0.321
Trelizumab	0.687	0.367-1.286	0.241	1.376	-0.375	0.320
Others	0.710	0.338-1.493	0.367	0.815	-0.342	0.379
Chemotherapy						
No	Ref					
Yes	1.376	0.882-2.149	0.160	1.976	0.320	0.227
Pathology						
Adenocarcinoma	Ref					
Small cell carcinoma	0.794	0.461-1.369	0.407	0.687	-0.230	0.278
Squamous cell carcinoma	1.763	0.859-3.620	0.122	2.385	0.567	0.367
Others	0.336	0.043-2.643	0.300	1.074	-1.091	1.052
Staging						
II-III	Ref					
IV	1.483	0.837-2.627	0.177	1.822	0.394	0.292
Efficacy assessment						
PD	Ref					
SD	0.733	0.461-1.163	0.187	1.739	-0.311	0.236
PR	0.355	0.175-0.719	0.004	8.253	-1.036	0.361
Lung metastasis						
No	Ref					
Yes	0.650	0.401-1.055	0.081	3.044	-0.431	0.247
Liver metastasis						
No	Ref					
Yes	2.058	1.111-3.812	0.022	5.268	0.722	0.314

HR, hazard ratio; CI, confidence interval; SE, standard error; Ref, reference; ICIs, immune checkpoint inhibitors; PD, disease progression; SD, stable disease; PR, partial remission.

### Construction and validation of nomograms

**Figure 3:**
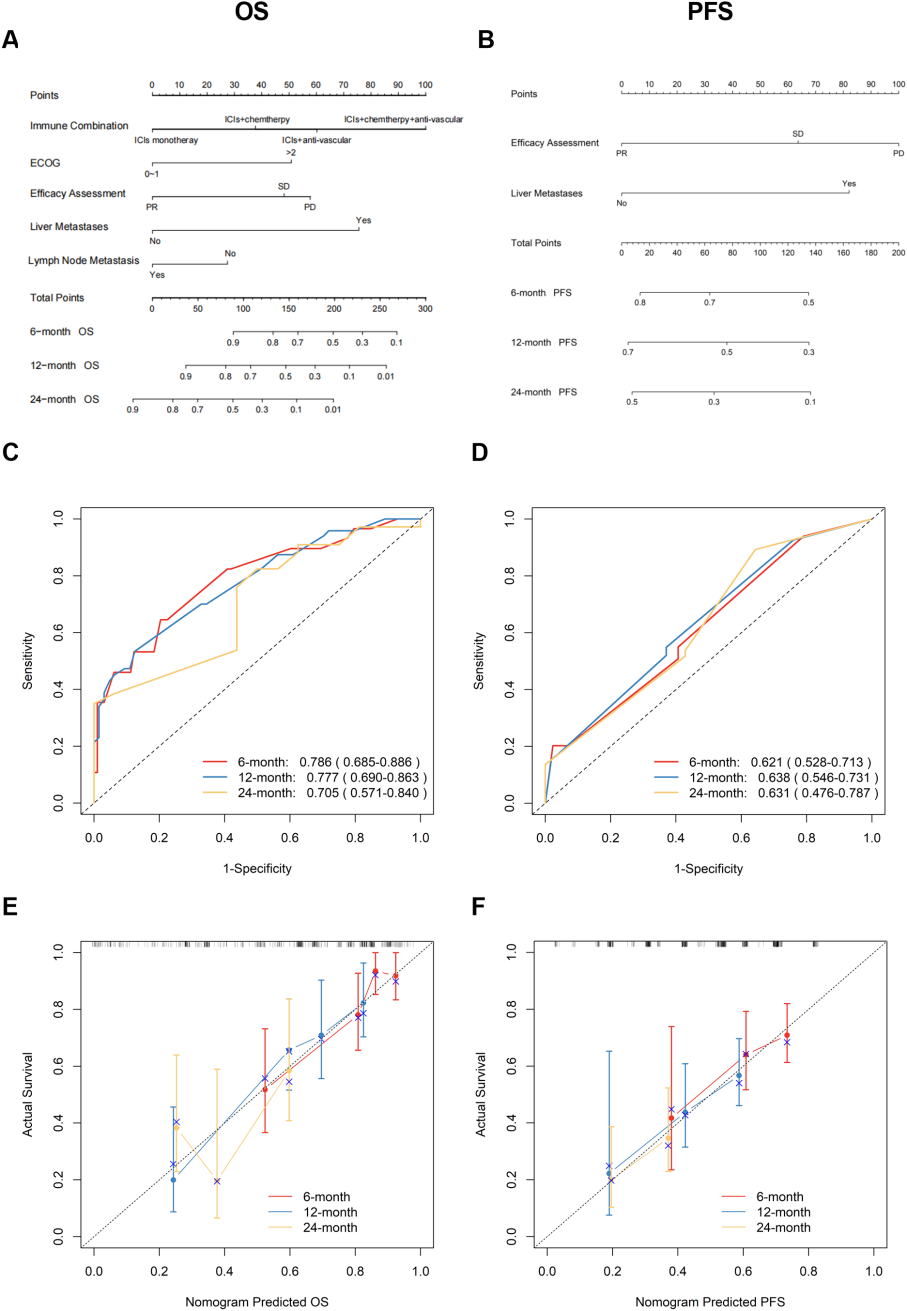
Development and validation of OS/PFS prediction nomograms. **(A)** OS and **(B)** PFS nomograms for predicting progression of ICIs therapy in patients with lung cancer; Time-dependent ROC curves and AUC (95% CI) for **(C)** OS and **(D)** PFS prediction; Calibration plots comparing predicted vs observed outcomes at 6/12/24 months for **(E)** OS and **(F)** PFS. OS, overall survival; PFS, progression-free survival; ICIs, immune checkpoint inhibitors; ROC, receiver operating characteristic; AUC, area under the curve; CI, confidence interval.

Based on multivariate Cox regression analysis, nomogram models were developed to predict 6-, 12-, and 24-month OS and PFS for patients. **Figure 3A** shows the independent predictors for OS, which include immunological combination regimens, ECOG performance status, efficacy assessment, and metastases in the liver and lymph nodes. **Figure 3B** illustrates the PFS prediction model, featuring efficacy assessment and liver metastasis as key factors. The ROC curve for the OS prediction model showed AUC values of 0.786, 0.777, and 0.705 at 6, 12, and 24 months, respectively. Similarly, the PFS model achieved AUC of 0.621, 0.638, and 0.631 at corresponding intervals (**Figures 3C, D**). The C-index values were 0.700 (95% CI: 0.636-0.772) for OS and 0.599 (95% CI: 0.535-0.662) for PFS, respectively. Calibration plots demonstrated a good agreement between the predicted and actual values for both the OS and PFS nomograms, showing no significant deviations (**Figures 3E, F**).

### Clinical applicability

**Figure 4:**
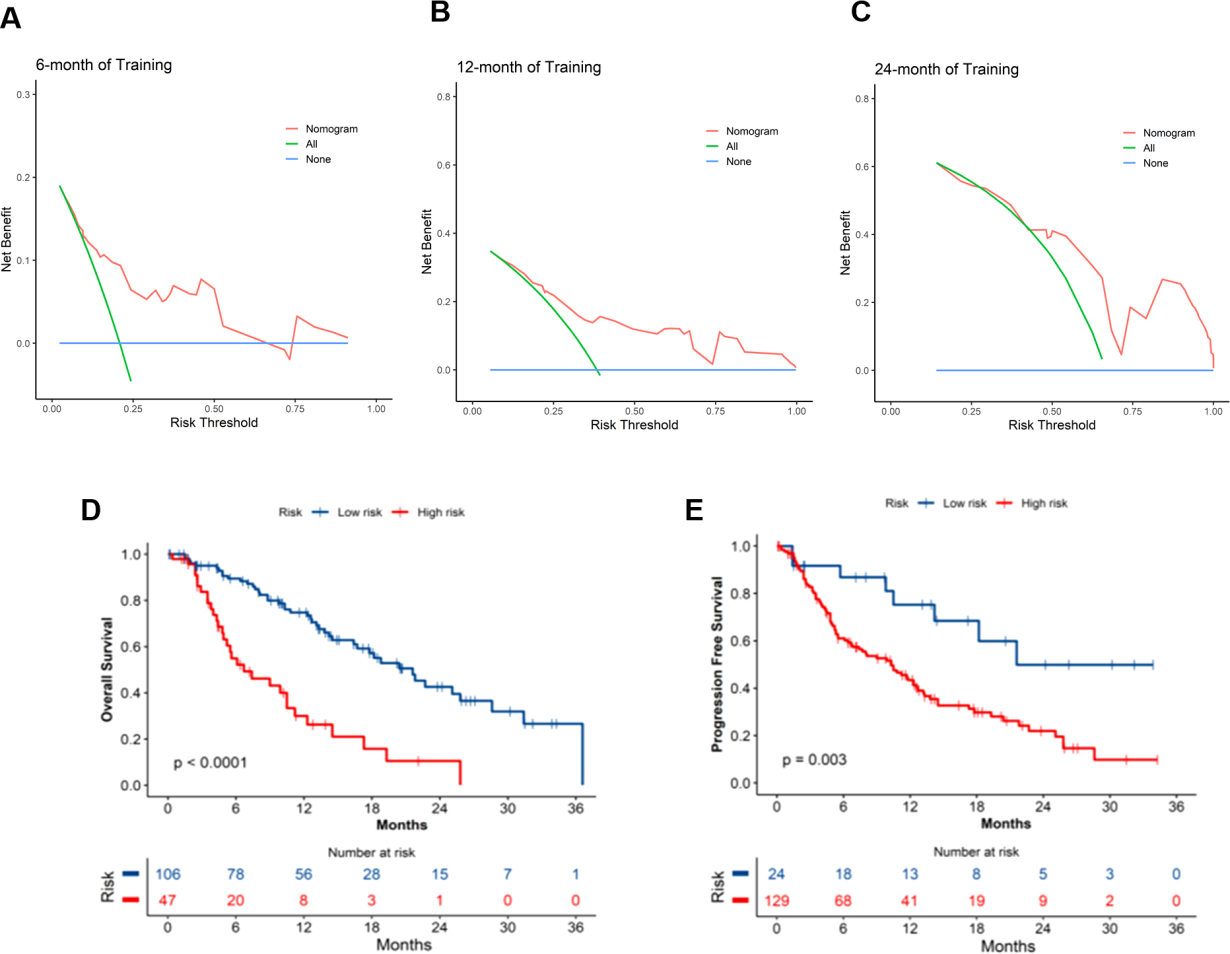
Clinical application of OS and PFS nomogram models. **(A-C)** DCA curves for OS nomogram at 6, 12, and 24 months; Survival curves for the high- and low- risk groups in the **(D)** OS and **(E)** PFS prediction models. OS, overall survival; PFS, progression-free survival; DCA, decision curve analysis.

The clinical applicability of the OS and PFS prediction models was rigorously assessed through DCA. As illustrated in **Figures 4A-C**, the OS nomogram demonstrated significant net clinical benefit across threshold probabilities. Using optimal cutoff values (OS: 129.02; PFS: 64.38), patients were stratified into distinct risk cohorts. Kaplan-Meier survival curves revealed marked disparities between groups, with the low-risk cohort achieving superior median OS (20.4 months vs. 6.7 months, p < 0.0001) and PFS (21.6 months vs. 10.3 months, p = 0.003), validating the models’ discriminative accuracy (**Figures 4D, E**). These results robustly confirm the prognostic validity and translational relevance of the nomograms.

## Discussion

This study developed and validated a clinicopathologic nomogram incorporating five independent prognostic factors—treatment regimen, ECOG performance status, RECIST v1.1-based efficacy evaluation, lymph node metastasis, and liver metastasis—to predict survival outcomes in advanced NSCLC patients receiving immune checkpoint inhibitors treatment beyond progression. The model demonstrated robust predictive performance, with a C-index of 0.700 (95% CI: 0.636–0.772) for OS and 0.599 (95% CI: 0.535–0.662) for PFS. It effectively stratified patients into high-risk (median OS: 6.7 months) and low-risk (median OS: 20.4 months, p < 0.01) subgroups, providing a quantifiable decision-making tool for patients with low PD-L1 expression or lacking actionable driver mutations, thereby addressing a critical unmet need in clinical practice.

The potential advantages of TBP: the investigators conducted various studies on atezolizumab in metastatic urothelial carcinoma, nivolumab in renal cell carcinoma and melanoma, and TBP involving anti-PD-1 drugs in metastatic non-small-cell lung cancer and melanoma (14–19). Our findings align with key clinical evidence: the phase III OAK trial demonstrated that NSCLC patients continuing atezolizumab post-progression achieved superior median OS compared to those switching therapies or discontinuing treatment (12.7 vs. 8.8 vs. 2.2 months), with a clinically meaningful 18-month OS rate of 37% and manageable safety (7). Consistent with this, a real-world study of 248 advanced lung cancer patients reported significantly prolonged OS (14.1 vs. 6.0 months, p = 0.028) and PFS (8.6 vs. 4.0 months, p = 0.028) in the TBP cohort, alongside a 5-fold higher objective response rate (12.1% vs. 2.4%) (20). However, conflicting evidence emerged from European and Japanese cohorts: no significant OS benefit was observed for TBP in PD-L1-high (≥ 50%) NSCLC patients receiving pembrolizumab or advanced NSCLC populations, though inflammation-related biomarkers (e.g., CRP, ALI) correlated with survival in the latter (11, 12). These discrepancies underscore the impact of patient heterogeneity on TBP outcomes.

Lymph nodes, as peripheral lymphoid organs, serve as critical hubs for lymphocyte proliferation and adaptive immune response initiation. Intriguingly, lymph node metastasis—traditionally a poor prognostic marker—emerged as a protective factor (HR = 0.538, 95% CI: 0.291–0.995, p=0.048), potentially attributable to enriched CD4+/CD8+ memory effector T cells in metastatic lymph nodes (13.3–14.6% higher than non-metastatic nodes), suggesting localized immune microenvironment remodeling may enhance systemic antitumor responses (21). In contrast, liver metastasis correlated with markedly worse outcomes (HR = 2.925, 95% CI: 1.360–6.290, p=0.006), aligning with established mechanisms of hepatic immune tolerance. Mechanistically, this immunosuppressive tumor microenvironment arises from N1-acetylspermidine efflux-dependent SRC signaling activation, which concurrently drives CCL1 macrophage polarization and CCR8 regulatory T-cell recruitment (22). Lung cancer-derived liver metastases may induce biliary obstruction and hepatic dysfunction, which compromise physiological reserve and diminish immunotherapy responsiveness (23). Strikingly, patients with liver metastases exhibited the shortest median survival (3 months) compared to those with bone or central nervous system involvement (24). Notably, TROP2-ADCs reprogram the tumor microenvironment by inhibiting macrophage and fibroblast recruitment, offering a potential strategy to overcome hepatic immune tolerance in combinatorial therapies (25).

While immunotherapy monotherapy demonstrates substantial efficacy in NSCLC patients with high PD-L1 expression (tumor proportion score ≥ 50%), its benefits in PD-L1-low populations mirror those of conventional chemotherapy (26–29). Critically, even among PD-L1-high patients, 29.8% exhibit suboptimal responses to monotherapy, underscoring the necessity for combinatorial strategies (30). Chemoimmunotherapy synergistically enhances antitumor activity through immunogenic cell death induction and immune escape pathway disruption (31, 32), while anti-angiogenic agents remodel the tumor microenvironment by suppressing immunosuppressive cells (e.g., MDSCs, Tregs) and promoting T-cell infiltration via vascular normalization (33, 34). Despite the preclinical promise of emerging approaches like radioimmunotherapy or dual checkpoint inhibition, their post-progression clinical utility remains undefined. Our data suggest that initial ICI monotherapy may optimize TBP patient selection, with sequential integration of chemotherapy or antiangiogenic regimens offering additional clinical gains. Further mechanistic studies are warranted to elucidate the immunological basis of these therapeutic hierarchies.

The nomogram developed in this study addresses a distinct clinical niche. Compared to Li et al.’s comprehensive model for treatment-naïve NSCLC patients (incorporating 11 variables, C-index=0.717), our model focuses specifically on the clinically debated scenario of treatment beyond progression (35). Using only 5 routine clinical indicators, it achieves comparable OS predictive performance (C-index=0.700). The model also incorporates a PFS dimension to identify subgroups likely to benefit from sustained immune exposure, thereby helping to avoid unnecessary treatment toxicity and economic burden. Notably, we reveal a protective effect of lymph node metastasis, contrasting sharply with the strong negative impact of liver metastasis. These findings reflect the influence of spatial heterogeneity in the immune microenvironment on TBP efficacy, a novel insight not captured by existing models (36). Unlike genomics-driven models (e.g., TIDE score), our nomogram offers distinct advantages. In terms of clinical accessibility, it relies on routinely available parameters (e.g., metastatic patterns), eliminating dependency on complex gene sequencing or specialized bioinformatics platforms, reducing per-assessment costs, and enabling real-time risk stratification in resource-limited settings, thereby supporting the widespread implementation of stratified immunotherapy management in primary care institutions. Regarding therapeutic guidance, high-risk patients (score > 129.02) may benefit from prioritized enrollment in clinical trials evaluating bispecific antibodies (e.g., TGF-β/PD-L1 inhibitors) or novel immune combinations; conversely, low-risk patients (score ≤ 129.02) can maintain standard-of-care regimens to avoid overtreatment toxicity. Considering clinical utility, decision curve analysis confirmed significant net benefit for OS prediction at 6–24 months, supporting its integration into TBP decisions. The visual nomogram output further enhances clinician-patient communication by facilitating transparent discussions of prognosis and treatment expectations, which may improve treatment adherence and patient satisfaction.

## Limitations

Firstly, the model’s ability to identify immunotherapy-resistant subgroups is constrained by the insufficient coverage of routine biomarker testing (e.g., PD-L1 expression and EGFR/ALK/ROS1 driver genes). The heterogeneity within PD-L1-negative populations encompassing distinct resistance mechanisms, such as T-cell exhaustion or antigen presentation defects, remains inadequately characterized. Secondly, differences in the immune microenvironment across lung cancer pathological subtypes significantly impact ICIs response patterns and prognosis: small cell carcinoma exhibits an “immune desert” phenotype (characterized by insufficient CD8+ T-cell infiltration and Treg enrichment), whereas tertiary lymphoid structures within lymph node metastases in adenocarcinoma may potentiate anti-tumor immunity (37, 38). Due to sample size limitations, this study was unable to conduct in-depth histology-stratified subgroup modeling.

Although the two-factor PFS model, incorporating liver metastasis and efficacy assessment, provides clinical convenience (median PFS 21.6 vs. 10.3 months, p <0.001), variability in imaging interpretation due to immunotherapy-specific progression patterns (e.g., pseudoprogression) underscores the utility of integrating the OS nomogram (C-index=0.700) for comprehensive clinical decision-making. Future research will prioritize the following directions: 1) Initiate a multicenter prospective cohort study to standardize the collection of irAE severity (CTCAE v5.0), post-progression treatment modifications, and treatment intensity parameters (relative dose intensity ≥80%; maintenance duration ≥3 months). Include Western populations to validate racial/regional generalizability and expand the external validation cohort. 2) Enlarge the sample size for the high-risk subgroup with liver metastasis (HR = 2.925) to investigate interactions between metastatic burden (e.g., oligometastatic vs. diffuse), anatomical location (portal vs. peripheral), and treatment response. Extend the follow-up duration to at least 2 years to evaluate the model’s long-term predictive performance. 3) Establish a standardized platform for monitoring dynamic biomarkers: circulating tumor DNA clearance rate, tumor mutational burden dynamics, and serum IFN-γ levels, addressing limitations of static baseline parameters. Incorporate TROP2 expression and TGF-β concentration monitoring to extend the model’s predictive coverage to emerging therapies (e.g., TROP2-ADCs).

## Conclusion

In summary, we developed and validated an effective nomogram model to predict OS and PFS at 6, 12, and 24 months after progression to continued immunotherapy in lung cancer patients. The model demonstrated robust discriminatory ability and high predictive accuracy, with calibration curves showing good agreement between predicted and observed outcomes. This tool enables risk stratification and personalized therapeutic decision-making by identifying patients most likely to derive clinical benefit from treatment beyond progression, thereby assisting to address a critical need in precision oncology.

## Data Availability

The original contributions presented in the study are included in the article/supplementary material. Further inquiries can be directed to the corresponding authors.
